# Comparison of Simple Drainage Versus Drainage With Tract Destruction in the Management of Pilonidal Abscess

**DOI:** 10.7759/cureus.91699

**Published:** 2025-09-06

**Authors:** Dawan J Hawezy

**Affiliations:** 1 Surgery, Faculty of General Medicine, Koya University, Koya, IRQ

**Keywords:** abscess, drainage, pilonidal, sinus, surgery

## Abstract

Introduction: Pilonidal disease is an acquired condition primarily affecting the gluteal cleft and sacrococcygeal area, often complicated by the formation of sinuses and abscesses. Despite multiple surgical options, there is no universally accepted standard treatment. This study aimed to compare the outcomes of simple drainage alone versus drainage combined with sinus destruction using the same incision technique. Methods: Between February 2019 and March 2024, a total of 480 patients with acute pilonidal abscesses received treatment. Of these, 235 underwent simple drainage (Group A), while 245 had drainage with sinus damage (Group B). All participants were followed for at least nine months, with recurrence rates recorded throughout. Statistical analysis was performed using SPSS version 28 (IBM Corp., Armonk, NY, USA). Results: In Group A, 131 patients (55.7%) experienced a recurrence, compared to only 29 patients (11.8%) in Group B. This difference is statistically significant (p < 0.001). The majority of recurrences occurred in younger, male, and overweight individuals. Conclusion: Removing the sinus tract during the same procedure as abscess drainage greatly lowers the chance of the abscess recurring, compared to drainage alone. Incorporating sinus destruction into the initial surgery might eliminate the need for subsequent operations for people with pilonidal abscesses.

## Introduction

Pilonidal disease (PD) is an acquired disorder affecting the gluteal cleft and sacrococcygeal area, characterized by the development of sinuses and abscesses associated with midline pits that trap hair and debris [1]. Most cases occur in the buttock and gluteal region, with rare reports of involvement in other locations, such as the scalp, axilla, groin, or interdigital spaces [2]. Risk factors include a hairy body, thick skin, overweight status (body mass index [BMI] > 25 kg/m²), a deep gluteal cleft, poor hygiene, prolonged sitting, and repeated chafing. During World War II, American military surgeons referred to PD as “Jeep seat” due to its increased prevalence among Jeep drivers. A positive family history is also recognised as a predisposing factor [3].

The global average incidence of PD is 26 cases per 100,000 people, with the highest prevalence reported in the Caucasus region, and it occurs three times more often in men than in women [4]. No precise data are available on the prevalence or incidence of PD among residents of the Kurdistan region in Iraq [5]. The disease ranges from asymptomatic pilonidal sinuses to active infection, and approximately half of patients present with acute infection [1,5]. Pilonidal sinus disease has been recognised for many years; its first formal description was by Abraham Wendell Anderson (1804-1876) in 1847, according to de Parades et al. [3].

Although many surgical techniques have been described for treating PD and pilonidal abscesses, including drainage and curettage, cryosurgery, Z-plasty, excision with secondary healing, vacuum-assisted closure, and modified lay-open procedures (incision, curettage, partial lateral wall excision, and marsupialization), the optimal treatment approach remains controversial [6]. Minimally invasive techniques, such as laser and endoscopic approaches, have been introduced to shorten healing time. Endoscopy appears effective for both the acute and chronic phases of PD [7].

No standardized surgical protocol exists to improve the management of pilonidal abscesses. All techniques carry a risk of treatment failure and recurrence. This study aims to evaluate a modified drainage technique for pilonidal abscesses that reduces the risk of recurrence and the need for subsequent surgery to address pilonidal sinus tracts.

The question is, does drainage with tract destruction lower recurrence rates compared to simple drainage in individuals with acute pilonidal abscess? We posited that the incorporation of tract destruction into normal drainage would markedly diminish recurrence rates in comparison to conventional drainage alone.

## Materials and methods

The study received approval from the Institutional Review Board (IRB) of the School of Medicine at Koya University on January 9, 2019, under the code 270/35. The committee verified that formal clinical trial registration was unnecessary.

We conducted a prospective comparison study at a private surgical clinic operated by a general surgeon in Koya city, Kurdistan Region, Iraq, from February 2019 to March 2024. The research involved 480 patients clinically diagnosed with acute pilonidal abscess.

Groups for the study and randomisation

Eligible patients were randomly assigned to two groups: Group A (n = 235) received incision and straightforward drainage, and Group B (n = 245) received incision and drainage along with sinus tract destruction via the same incision, employing a mosquito clamp to dismantle tracts and eliminate leftover hair.

Criteria for inclusion and exclusion

Patients having an acute pilonidal abscess were incorporated. The exclusion criteria included reluctance to participate or accept local anesthetic, a previous history of pilonidal illness, chronic comorbidities, the existence of secondary openings, or more than five primary openings. Patients who did not attend their planned follow-up visits were also eliminated.

Surgical procedure

Both groups had the treatment done in a clean, sterile environment with local anesthetic. A No. 11 scalpel blade was used to make an incision off the midline and drain the abscess. In Group B, following drainage, a mosquito clamp was inserted into the incision toward the midline to break up sinus tracts. Before the wound was cleaned and dressed, any remaining hairs were removed. All patients were given antibiotics and painkillers for 10 days. Patients, surgeons, and outcome assessors were not blinded, which could lead to bias.

Follow-up and results

Follow-up evaluations were planned for three days, two weeks, and four weeks, then every three months for a year, and then every year after that. Before the data analysis, all patients were called and reviewed. Those who were lost to follow-up were not included. The trial guaranteed a minimum follow-up period of nine months.

Sample size and statistical analysis

The final analysis comprised 480 patients, with 235 in Group A and 245 in Group B. We used SPSS version 28 (IBM Corp., Armonk, NY, USA) to analyze the data and calculate p-values to see if the results were statistically significant.

## Results

A total of 480 patients completed a follow-up period of at least nine months. Of these, 235 patients (49%) in Group A underwent only simple drainage, while 245 patients (51%) in Group B underwent drainage with tract destruction. A majority of participants (303 patients, 63.1%) were male, aged between 16 and 43 years. Most patients (341, 71%) were between 20 and 29 years old. No significant differences were observed in age, sex, or body weight between the two groups. These demographic details are summarised in Table 1.

**Table 1:** Demographic distribution of the participants according to groups (N=480) *Calculated using the Pearson chi-square test (χ²).

Variable		Group A, n = 235, n (%)	Group B, n = 245, n (%)	Total, n (%)	P-Value*
Sex	Male	142 (60.4%)	161 (65.7%)	303 (63.1%)	0.256
	Female	93 (39.6%)	83 (33.9%)	177 (36.9%)	
Age (years)	16-19	64 (27.2%)	57 (23.3%)	121 (25.2%)	0.388
	20-29	160 (68.1%)	181 (73.9%)	341 (71.0%)	
	30-40	10 (4.3%)	7 (2.9%)	17 (3.5%)	
	More than 40	1 (0.4%)	0 (0.0%)	1 (0.2%)	
Body mass index, kg/m^2^	Healthy (18.5-24.9)	92 (39.1%)	109 (44.5%)	201 (41.9%)	0.154
	Overweight/Obese (>24.9)	117 (49.8%)	101 (41.2%)	218 (45.4%)	
	Underweight/Thin (<18.5)	26 (11.1%)	35 (14.3%)	61 (12.7%)	

**Figure 1:**
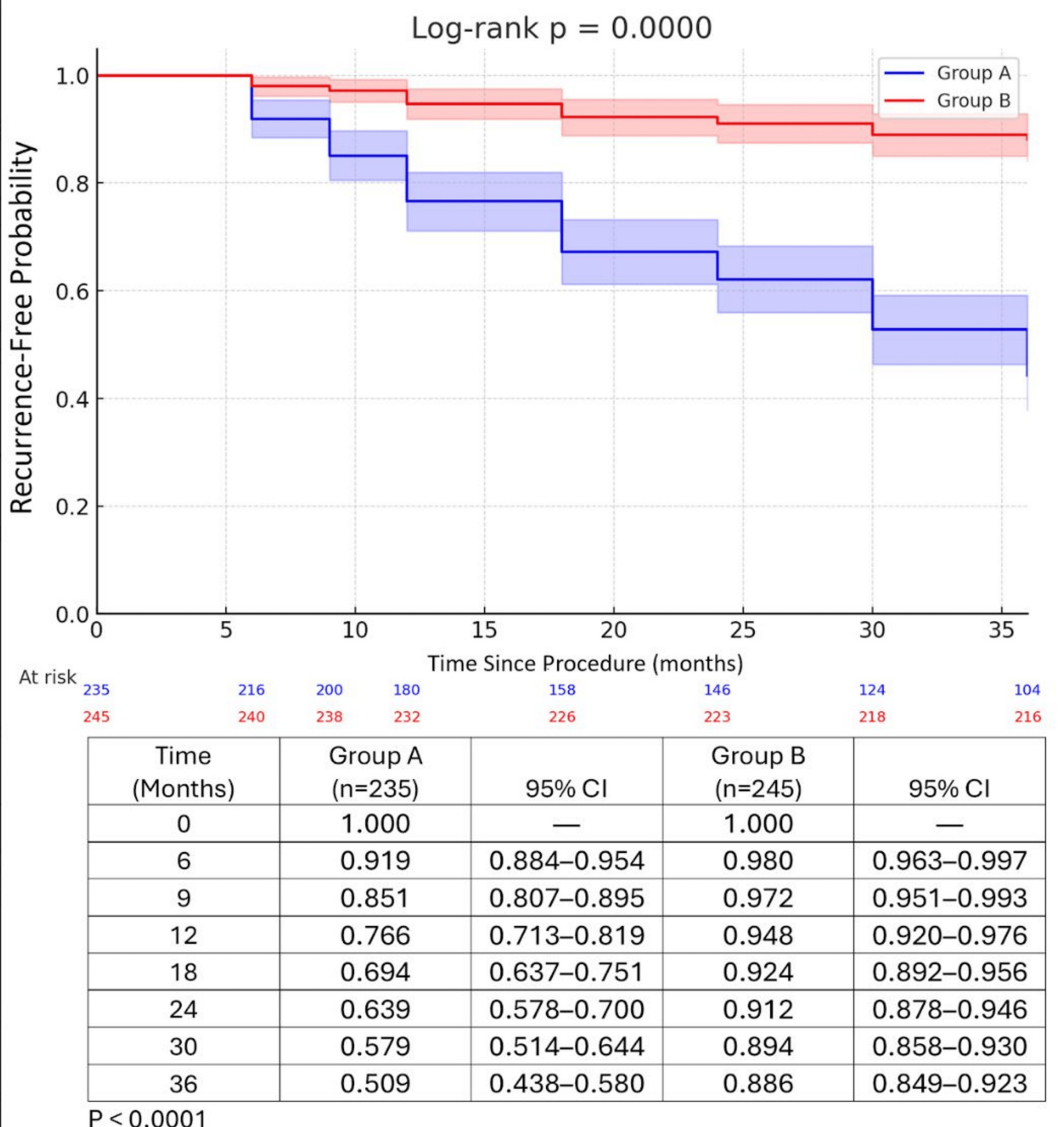
Kaplan-Meier curve to show the recurrence of both groups p value less than 0.0001

Recurrence or persistence of a pilonidal sinus occurred in 131 patients in Group A and 29 patients in Group B. Table 2 provides details of these findings. We followed up with all patients and contacted them before study completion. Patients who could not be examined in person were excluded from the study. The Kaplan-Meier curve in Figure 1 illustrates the recurrence rates for both groups.

**Table 2:** Recurrence or existence of pilonidal abscess in both groups *Calculated using the Pearson chi-square test (χ²).

Recurrence Variable	Group A, n = 235, n (%)	Group B, n = 245, n (%)	Total, n (%)	P-Value*
Recurrence or existence	131 (55.7%)	29 (11.8%)	160 (42%)	< 0.001
No recurrence	104(44.2%)	216 (88.1%)	278 (57.9%)	
Total	235 (100%)	245 (100%)	480 (100%)	

We observed notable differences in occurrence and recurrence rates between males and females. In Group B, only eight of 83 females experienced recurrence. Table 3 presents these differences, while Table 4 shows age-related outcomes, indicating that 164 of 181 patients aged 20 to 30 years had no recurrence.

**Table 3:** Sex difference in the occurrence and recurrence of pilonidal abscess *Calculated using the Pearson chi-square test (χ²).

Sex	Procedure	No Recurrence, n	Recurrence or Existence, n	Total, n
Female	Simple drainage	35	58	93
	Destruction of the tract	76	8	84
Male	Simple drainage	69	73	142
	Destruction of the tract	140	21	161
P-Value*		0.787	0.99	0.23

**Table 4:** Age difference in the occurrence and recurrence of pilonidal abscess *Calculated using the Pearson chi-square test (χ²).

Age in Years	Procedure	Recurrence, n		Total, n	P-Value*
		No	Yes		
16-19	Simple Drainage	29	35	64	0.002
	Destruction of the tract	45	12	57	
	Total	74	47	121	
20-29	Simple Drainage	70	90	160	<0.001
	Destruction of the tract	164	17	181	
	Total	234	107	341	
30-40	Simple Drainage	5	5	10	0.44
	Destruction of the tract	7	0	7	
	Total	12	5	17	
Older than 40	Simple Drainage	0	1	1	<0.001
	Destruction of the tract	0	0	0	
	Total	0	1	1	
Total		320	160	480	

A total of 188 patients were classified as overweight or obese based on their BMI. Of these, 107 patients experienced recurrence despite undergoing either type of surgical intervention. Table 5 provides further details.

**Table 5:** Obesity difference in the recurrence of pilonidal abscess *Calculated using the Pearson chi-square test (χ²).

Bodyweight Classification	Procedure	Recurrence, n		Total, n	P-Value*
		No	Yes		
Healthy	Simple drainage	49	43	92	<0.001
	Destruction of the tract	108	1	109	
	Total	157	44	201	
Overweight/obese	Simple drainage	37	80	117	0.001
	Destruction of the tract	74	27	101	
	Total	124	107	188	
Underweight/thin	Simple drainage	18	8	26	0.322
	Destruction of the tract	34	1	35	
	Total	52	9	61	
Total	Simple drainage	104	131	235	<0.001
	Destruction of the tract	216	29	245	
	Total	320	160	480	

## Discussion

Although incision and drainage of a pilonidal abscess often achieve high initial success rates, recurrence remains a substantial problem. Previous studies report success rates of 70%-80%, yet up to 40% of patients experience recurrence within one to two years [8]. More recent evidence suggests that success rates can approach 90% with meticulous wound care and follow-up; however, long-term rates often decline to 60%-70% due to recurrence [9]. Our results for Group A, in which nearly half of the patients experienced recurrence after simple drainage, mirror these long-standing concerns and emphasise the need for more effective surgical strategies.

In response to the limitations of standard drainage, various modified techniques have been developed to reduce recurrence. Even for pilonidal sinus without abscess, newer approaches such as endoscopic pilonidal sinus treatment and laser ablation have shown high initial success but still report some failures [10,11]. In our study, adding tract destruction at the time of drainage achieved a lower recurrence rate than simple drainage alone, and the results compare favorably with other published strategies, including one that reported a higher recurrence after a similar intervention in Tunis [12].

Recurrence in our cohort was more frequent in male patients, supporting prior evidence that anatomical differences, hair density, and lifestyle factors may contribute to a higher risk in this group [13,14]. Younger patients also had higher recurrence rates, which may relate to increased physical activity and denser hair growth, consistent with earlier findings [9,13,15,16]. Overweight and obese patients remained at elevated risk regardless of surgical method, likely due to factors such as a deeper gluteal cleft, impaired wound healing, increased wound tension, and greater friction [14,17].

Limitations

There are several issues with this study. One surgeon at one centre did it first, which may limit its usefulness elsewhere. Second, not blinding either the patients or the outcome assessors could lead to observer or performance bias. Third, the study compared simple drainage with drainage plus sinus tract destruction. Still, it did not compare these methods to other sophisticated or minimally invasive treatments (e.g., endoscopic or laser approaches), which limits the conclusions that may be drawn. Fourth, despite all patients having a minimum follow-up of nine months, this period may be inadequate to detect very late recurrences that can arise years post-surgery. Furthermore, significant unmeasured confounders-such as patients’ occupation, daily sitting duration, personal hygiene habits, and socioeconomic status-were not comprehensively evaluated. It is known that these factors can affect both the onset and recurrence of pilonidal disease. Not including them in our analysis may have changed the patterns of recurrence we saw. These limitations indicate that, although the findings reveal a substantial decrease in recurrence with tract destruction, one should be prudent in extrapolating the results to larger populations or diverse clinical contexts. Subsequent multicenter, randomized controlled trials with extended follow-up and a more thorough evaluation of patient-related risk variables are necessary to validate and enhance these findings.

## Conclusions

This study evaluated whether adding tract destruction to standard drainage for acute pilonidal abscess could reduce recurrence and eliminate the need for a subsequent operation to remove the sinus tract. The results showed that this modified technique was associated with fewer recurrences than simple drainage alone, with benefits observed across different patient groups. Although certain risk factors, such as younger age and higher BMI, remained associated with recurrence, the addition of tract destruction improved outcomes in all subgroups. By reducing the likelihood of repeat surgery, this approach offers a practical and effective refinement to current management practices, with the potential to lessen patient morbidity, shorten recovery, and lower the overall burden of care in the treatment of PD.
